# AI-based digital histopathology for perihilar cholangiocarcinoma: A step, not a jump

**DOI:** 10.1016/j.jpi.2023.100345

**Published:** 2023-11-05

**Authors:** Dieter P. Hoyer, Saskia Ting, Nina Rogacka, Sven Koitka, René Hosch, Nils Flaschel, Johannes Haubold, Eugen Malamutmann, Björn-Ole Stüben, Jürgen Treckmann, Felix Nensa, Giulia Baldini

**Affiliations:** a University Hospital Essen, Department of General, Visceral and Transplantation Surgery, Essen, Germany; b University Hospital Essen, Institute for Pathology and Neuropathology, Essen, Germany; c Institute of Pathology Nordhessen, Kassel, Germany; d University Hospital Essen, Institute of Interventional and Diagnostic Radiology and Neuroradiology, Essen, Germany; e University Hospital Essen, Institute for Artificial Intelligence in Medicine, Essen, Germany

**Keywords:** Klatskin, Cholangiocarcinoma, Survival analysis, Segmentation, Digital pathology, Artificial intelligence

## Abstract

**Introduction:**

Perihilar cholangiocarcinoma (PHCC) is a rare malignancy with limited survival prediction accuracy. Artificial intelligence (AI) and digital pathology advancements have shown promise in predicting outcomes in cancer. We aimed to improve prognosis prediction for PHCC by combining AI-based histopathological slide analysis with clinical factors.

**Methods:**

We retrospectively analyzed 317 surgically treated PHCC patients (January 2009–December 2018) at the University Hospital of Essen. Clinical data, surgical details, pathology, and outcomes were collected. Convolutional neural networks (CNN) analyzed whole-slide images. Survival models incorporated clinical and histological features.

**Results:**

Among 142 eligible patients, independent survival predictors were tumor grade (G), tumor size (T), and intraoperative transfusion requirement. The CNN-based model combining clinical and histopathological features demonstrates proof of concept in prognosis prediction, limited by histopathological complexity and feature extraction challenges. However, the CNN-based model generated heatmaps assisting pathologists in identifying areas of interest.

**Conclusion:**

AI-based digital pathology showed potential in PHCC prognosis prediction, though refinement is necessary for clinical relevance. Future research should focus on enhancing AI models and exploring novel approaches to improve PHCC patient prognosis prediction.

## Highlights

• Convolutional neural networks can create heatmaps for perihilar cholangiocarcinoma.
• The generated heatmaps could aid pathologists in identifying areas of interest.
• A survival analysis using model features showed limited clinical relevance.
• Tumor grading, tumor size, and intraoperative transfusion were significant factors.

## Introduction

Perihilar cholangiocarcinomas (PHCC), also known as Klatskin carcinoma, are rare with an incidence of 1–2 cases per 100 000 in the western world. It is characterized by late diagnosis, complex surgical treatment, and a severely limited prognosis: dismal 5-year survival rates between 13.5% and 42% have been reported even for surgically curatively treated cases.^1^, ^2^, ^3^, ^4^ Individual, prognosis-driving factors besides tumor characteristics are hardly known. Several studies have evaluated predictive models for an individual survival prognosis of the patients.^5^, ^6^, ^7^, ^8^, ^9^, ^10^, ^11^ Unfortunately, individual prognosis prediction is difficult with the current level of knowledge available.

In the recent past, artificial intelligence-based digital pathology analysis demonstrated outstanding results for the prediction of survival in several oncological entities.^12^, ^13^, ^14^ Applying this new technology to perihilar cholangiocarcinoma yields the promise to identify histological features that can be correlated with the clinical outcome. The identification of subtypes is a specific goal to allow an improved prognosis prediction on an individual basis in the context of the overall low survival rate for the Klatskin carcinoma population.

Therefore, we aimed to identify predictive clinical factors in a monocenter cohort of patients with perihilar cholangiocarcinoma and combine these with results of AI-based analysis of histopathological slides for an improved prediction of prognosis.

## Material and methods

### Study design

We performed a retrospective analysis of all patients who underwent surgical treatment for perihilar cholangiocarcinomas between January 2009 and December 2018 at the University Hospital of Essen. Data of all patients (*n* = 317) with the diagnosis of a perihilar cholangiocarcinoma were extracted from the digital hospital information system. Two independent investigators verified each patient's diagnosis. Only patients treated surgically with complete histopathological investigation were included in this study.

This retrospective study was approved by the local ethics committee (19-8681-BO) and followed the Declaration of Helsinki.

### Data

The following data were extracted for each patient from the digital hospital information system:

Preoperative clinical data: Age, sex, BMI, preoperative biliary stenting, preoperative cholangitis, preoperative laboratory values (bilirubin, INR, creatinine, platelets, gGT, and CA19-9), and preoperative MELD score.

Surgery: Type of resection, operative time, blood transfusion, portal vein reconstruction, and hepatic artery reconstruction.

Postoperative pathology: TNM classification (including lymph node count, lymph node status, and perineural invasion), Union for International Cancer Control (UICC) classification, tumor size, and histological subtype.

Postoperative clinical data: Duration of ICU stay, duration of hospital stay, in-hospital mortality, laboratory values on postoperative day (POD) 5 (bilirubin, International Normalized Ratio (INR)), postoperative complications (hepatic failure, biliary leakage, abscess, bleeding, relaparotomy, and kidney failure), and overall survival.

We did not include adjuvant treatments (radiotherapy, chemotherapy) in the data because the period under investigation lies mostly before the publication of the BILCAP^15^ study when adjuvant treatments were not routinely established after the resection of biliary tract cancers. All patients were postoperatively discussed in the interdisciplinary tumor board giving recommendations on the further clinical pathway. As such, patients presumably had fairly uniform recommendations leading to uniform postoperative treatments. However, patients were followed at several different oncological treatment centers, which led to data incompleteness on adjuvant therapies.

### Definitions

#### MELD score

The MELD score^16^ (Model for End-stage Liver Disease) indicates the severity of liver disease. The MELD score is calculated from bilirubin, creatinine, and INR (International Normalized Ratio) as follows:

9.57×ln(serumcreatinine[mg/dL])+3.78×ln(totalbilirubin[mg/dL])+11.2×ln(INR)+6.43

If dialysis was performed within the week before the examination, the creatinine value was set to 4.0.

#### Postoperative liver failure

The International Study Group of Liver Surgery (ISGLS) defined PHLF as a postoperative acquired deterioration in the ability of the liver to maintain its synthetic, excretory, and detoxifying functions, which are characterized by an increased INR and concomitant hyperbilirubinemia on or after POD 5.^17^

### Surgery

Most patients underwent preoperative biliary drainage with the goal of reducing cholestasis to a serum total bilirubin level below 5 mg/dL.

Surgical treatment consisted of resection of the extrahepatic bile duct, intraoperative histopathology by frozen biopsies, and eventual extension of the resection as a pancreatoduodenectomy and/or liver resection. Liver resections were usually carried out as (extended) hemihepatectomy. All lymphatic and soft tissues were resected within the ligamentum hepatoduodenale. Few patients underwent portal vein or hepatic artery reconstruction due to cancer infiltration.

### Digital pathology

All clinical slides were reviewed by an experienced hepatobiliary pathologist. Hematoxylin and eosin stained slides were scanned using the Scanner Aperio® AT2 (Leica Biosystems, Wetzlar, Germany). Whole-Slide Imaging (WSI) was performed using a 40× objective lens with a maximal scanning resolution of 0.25 μ/pixel. Scanned images in the SVS file format were re-reviewed by an experienced hepatobiliary pathologist on a high-definition resolution screen (1930 × 1080 pixels). Images and data were stored and exported to an external storage device.

In every slide all tumor islands, nodules, and nests with at least 2 tumor cells were marked with the software QuPath.^18^ Areas of necrosis, crush artifacts, or irregular H&E staining were excluded.

### CNN-based slide analysis

The annotated WSIs were postprocessed to set the annotated areas to “tumor” and the non-annotated areas to “non-tumor”, resulting in a binary representation of the WSI. To make the WSIs easier to process, they were converted into patches. To ensure the usage of meaningful patches, an Otsu thresholding was performed, and only patches containing less than 50% background were selected.

**Fig. 1:**
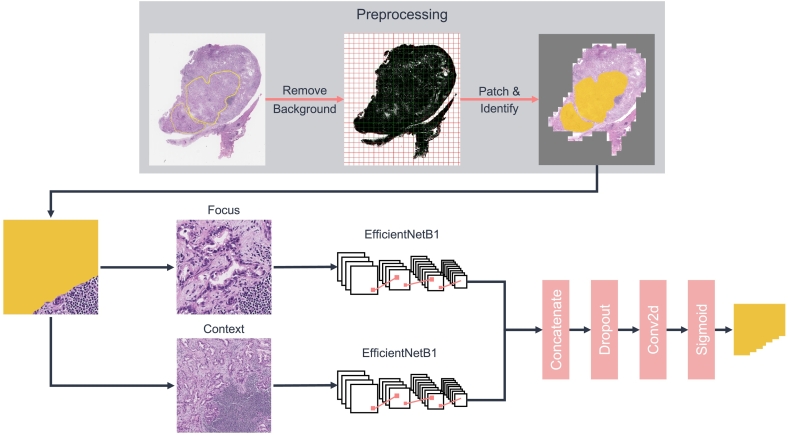
Preprocessing and training process. Each whole-slide image is first preprocessed by removing the background and by patching. The patches are used to train a double-pathway convolutional neural network using a high resolution patch as focus and a context patch. The model is trained to compute a segmentation of typical Klatskin tumor areas.

A double pathway EfficientNetB1^19^ convolutional neural network (CNN) was employed for the analysis of histopathology images. For training, a dataset of 85 whole-slide images (WSI) and converted into patches using 2 different resolutions (20× and 5×), resulting in 112 443 20× patches and 112 443 respective 5× patches. The network was designed to obtain a high and a low resolution patch as input corresponding to the same area, emulating the pathologist's approach, who assesses both cell morphology and surrounding structures. Data augmentation techniques such as horizontal and vertical flips, rotation, brightness, and contrast adjustments were used on the patches to improve the generalizability of the network. The patches were processed through 2 distinct EfficientNetB1 networks, concatenated, and converted into a segmentation mask of typical Klatskin tumor areas, as presented in Fig. 1. The output of the model is a mask of probabilities, and a threshold of 0.5 was used to consider a pixel as tumor.

During training, the model was first initialized with pre-trained ImageNet^20^ weights and then fine-tuned on this dataset. Since 80% of the ground-truth pixels were classified as non-tumor, class weights were used to account for the imbalanced nature of the dataset. The Adam optimizer was used with a learning rate of 0.0001 that decayed over time. The model was trained for 11 epochs during transfer learning and 120 epochs during fine-tuning together with early stopping technique to prevent overfitting. The training was performed using 5-fold cross-validation and each training run had 68 WSI for training and 17 WSI for validation. The number of patches per cross validation fold was however different: 30 974 for the first fold, 22 942 for the second, 15 388 for the third, 17 500 for the fourth and 25 639 for the fifth.

The preprocessing was computed with an in-house extension of the py-wsi package.^21^ The models were built using Python 3.8 and TensorFlow 2.6.2.^22^

### Survival model

In the survival prediction analysis, a total of 142 WSI (142 patients) were used. A held-out test set of 27 patients was used to evaluate the model, while the remaining 115 patients were used for training and validation with bootstrapping. The trained CNN network was utilized to evaluate and extract CNN features from patches that were identified as tumor by the pathologist. The features were extracted after the concatenation layer of each cross-validation model, resulting in 2560 features per cross-validation model, for a total of 12 800 features per patch. Given the potential for redundancy in these features, a data reduction step was implemented. The features were grouped using k-means clustering^23^ (using Rapids cuml^24^ version 22.02) with 500 clusters and normalized using the term frequency-inverse document frequency (TF-IDF) method.^25^ Different numbers of clusters (5, 10, 50, 100, 200, 500, 1000, 2000, 3000, and 5000) were tested using the Elbow method,^26^ and 500 was found to be the most appropriate in terms of computational speed and inertia. This resulted in obtaining a feature vector of length 2500 for each WSI. To reduce the dimensionality of the feature vectors, the non-negative matrix factorization method^27^ from scikit-learn (version 1.0.2)^28^ was employed, which resulted in 5 final features. Finally, the resulting features were used to train a gradient-boosted regression tree with Cox loss from scikit-survival^29^ (version 0.17.2). To ensure the statistical validity of the results, the process was repeated with 1000 rounds of bootstrapping.

**Fig. 2:**
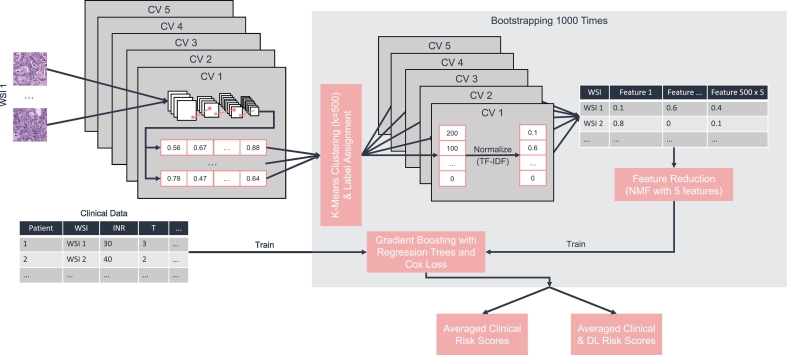
Process to generate risk scores for survival analysis. Each whole-slide image (WSI) is processed through the 5 cross-validation (CV) models, and features are extracted. The features are clustered using k-means clustering into 500 clusters, each feature is assigned to a cluster and the result is normalized using the term frequency-inverse term frequency (TF-IDF) method. The features from the 5 models are collected into a single feature vector for each WSI. Finally, feature reduction is applied, and the resulting features are used for training a gradient-boosted regression tree. Either the deep learning features are either used, or the clinical features, or a combination of both.

For the machine learning survival analysis, 2 different evaluations were conducted: 1 with clinical features, and 1 with a combination of clinical and deep learning features (see Fig. 2). The risk scores for each model were averaged over 1000 rounds of bootstrapping to improve statistical validity. For each model a hyperparameter optimization was run with optuna (version 2.10.0)^30^ and the hyperparameters with the best average concordance index were chosen.

Imputation was performed for missing values in the clinical features, Table S1 of the supplementary material reports both the variables subject to imputation and the corresponding count of missing data points. The data points were missing at random. The median was taken for variables such as CA199, LDH, weight, height, and days between first diagnosis and operation. Variables such as performed biliary stent, perineural invasion, UICC stadium, spread to the blood vessels, spread to the lymphatic vessels, and T grading were substituted with 0, while the G grading was substituted with 2, which is the most common grading for this tumor.

### Statistics

Normality was tested using the Shapiro–Wilk test. Survival analysis was performed by Kaplan–Meier statistics and compared by log-rank tests. We performed univariable cox regression analysis to identify predictors of patient survival. All factors with statistical significance were included in a multivariable cox regression model (conditional backwards selection). A *P*-value of <.05 was considered statistically significant. Data were analyzed using SPSS 27.0 software (IBM Inc., Armonk NY, USA). Data are given as mean values with standard deviation or with median and range as appropriate.

The CNN models were evaluated using accuracy, specificity, sensitivity/recall, precision, and F1-score. 95% confidence intervals were computed using the Clopper–Pearson exact method. The survival models were evaluated by taking the average risk scores and splitting them at the median. The 2 groups were used to build Kaplan–Meier plots and compared by log-rank tests. The concordance index was also computed for each model. Additionally, the impurity-based feature importance^31^ of the clinical features was analyzed.

## Results

### Study population

**Fig. 3:**
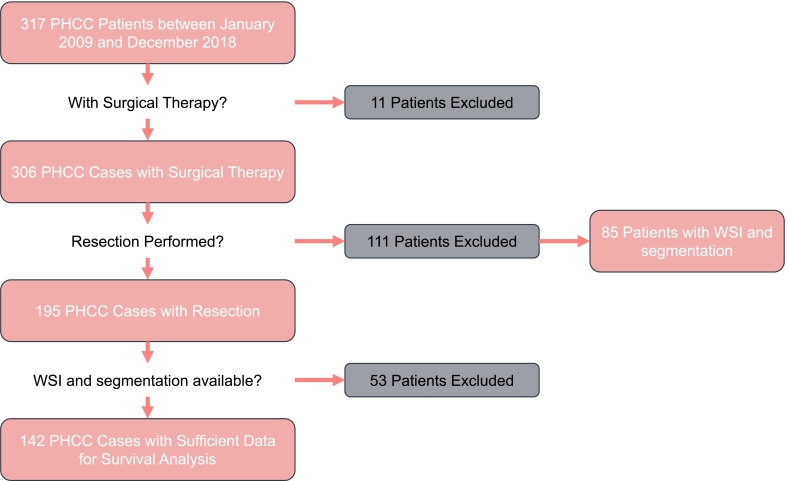
Flowchart of the cases. After the exclusion of PHCC cases without surgical therapy, only those with explorative surgery were considered. The survival analysis was performed on 142 cases that had clinical data, WSIs, and corresponding segmentations. Additionally, 85 WSI scans collected during exploratory surgery could be utilized to train a general CNN network for the generation of Klatskin heatmaps.

We treated 317 patients for Klatskin carcinoma at our department during the study period. Several patients were excluded: surgical therapies were not applied in 11 patients, 111 patients were explored and found to be unresectable. In total, 142 cases qualified for whole-slide image analysis and had sufficient clinical data. Therefore, these cases were utilized for survival analysis. Of the other patients, 85 had a WSI with a segmentation that could be used, but did not have sufficient clinical data to be included in the survival analysis. The flowchart of the cases is presented in Fig. 3.

### Demographics

In total, 142 patients data qualified for survival analysis and are characterized here. At the time of surgery, the mean age was 66.5 ± 9.2 years. The age of patients undergoing surgery for Klatskin tumor ranged from 36 to 82 years. Patients were predominantly male (100 (70.4%)), with a mean body height of 172 ± 8.8 cm and a mean body weight of 77.8 ± 15 kg. The resulting BMI was 26.02 ± 4.1 kg/m^2^ (range 15–40 kg/m^2^). The diagnosis was specified median 29 (7–174) days before surgery. Most patients (102 (71.8%)) were treated by preoperative biliary drainage. The majority of patients had comorbidities with preoperative ASA-Stage 3 in 60 (42.3%) patients and ASA-Stage 2 in 34 (23.9%) patients. Preoperative laboratory values are given in Table 1.

**Table 1:** Preoperative laboratory values. For each laboratory value, the median and the range was given, as all values were non-normally distributed according to the Shapiro–Wilk normality test. The standard values and the number of patients with non-standard values are also reported. The CA19-9 (U/l) laboratory value was only available for 63 patients.

Laboratory value	Median	Range	Standard values	Number and percentage of patients with non-standard values
Bilirubin (mg/dL)	1.2	0.2–20.6	≤1.1 mg/dL	73 (51.41%)
gGT (U/L)	393	13–3094	(m) ≤55 U/L (f) ≤38 U/L	(m) 99 (99%) (f) 38 (90.48%)
Creatinine (mg/dL)	1.01	0.47–2.85	(m) ≤1.1 mg/dL (f) ≤0.9 mg/dL	(m) 76 (76%) (f) 7 (16.67%)
INR	1	0.83–2.3	0.7–1.2	4 (2.82%)
Platelets (/nL)	294	16–714	130–280 /nL	80 (56.34%)
CA19-9 (U/L)	86.9	1–24 734	≤37 U/L	45 (71.42%)

### Surgery

The goal of surgery was an R0 resection. 37 (26.1%) patients were treated by en bloc resection of extrahepatic bile ducts. Additional (left or right) hemihepatectomy was performed in 50 (35.2%) patients and extended (left or right) hemihepatectomy in 29 (20.4%) patients. Pancreatoduodenectomy was performed in 26 (18.3%) patients. Portal vein or hepatic artery reconstruction was necessary in 15 (10.6%) patients and 1 (0.7%) patient, respectively. Transfusion of packed RBCs was performed in 14 (9.9%) patients. The median duration of surgery was 292 (98–567) min.

### Postoperative tumor characteristics

All resected specimens were sent to pathology and classified according to TNM and UICC classification. All UICC stages were reviewed and re-classified according to the current classification (8th edition) for better comparability. All tumor characteristics are given in Table 2, Table 3.

**Table 2:** Tumor characteristics (TNM classification) for 142 patients. T1–4 represents the T stadium, which was available for 139 patients (3 missing). G1–3 represents the tumor grading, which was available for 137 patients (5 missing). N−/+ represents whether the tumor spreaded to the lymphnodes. L0/1 represents whether the tumor spreaded to the lymphatic vessels, which was available for 133 patients (9 missing). V0/1 represents whether the tumor invaded the blood vessels, which was available for 132 patients (10 missing). Pn0/1 represents whether the tumor invaded the perineural sheath, which was available for 101 patients (41 missing). R0–2 represents the resection status, which was available for 130 patients (12 missing).

TNM classification	Number of patients	Percentage of patients (%)
T1	13	9.2
T2	82	57.7
T3	41	28.9
T4	3	2.1
G1	5	3.5
G2	98	69
G3	34	23.9
N−	59	41.5
N+	83	58.5
L0	109	76.8
L1	24	16.9
V0	121	85.2
V1	11	7.7
Pn0	19	13.4
Pn1	82	57.7
R0	77	54.2
R1	48	33.8
R2	5	3.5

**Table 3:** UICC stage classification (8^th^ Edition) for 112 patients, for the remaining 30 patients the UICC stadium was not available.

UICC stage classification (8^th^ Edition)	Number of patients	Percentage of patients (%)
1	4	2.8
2	34	23.9
3	71	50
4	3	2.1

### Postoperative complications & survival

Postoperatively, 59 (41.5%) patients developed liver failure as defined by the ISGLS. The following complications were recorded: bile leakage was observed in 23 (16.2%) cases, postoperative bleeding in 5 (3.5%) cases, and superinfected fluid collections/abscesses requiring treatment in 17 (12%) cases. Overall, 32 (22.5%) patients needed relaparotomy as part of the complication management strategy. During the initial postoperative hospital stay for surgery for Klatskin carcinoma, 25 (17.6%) patients died. The overall survival for patients undergoing resection for Klatskin carcinoma was 60.3%, 19.4%, and 7.8% after 1, 3, and 5 years, respectively. After censoring for in-hospital mortality and unsuccessful resections (R2-resection), the 1-, 3-, and 5-year survival rates were 75.4%, 24.3%, and 11.1%, respectively.

### Digital pathology

**Fig. 4:**
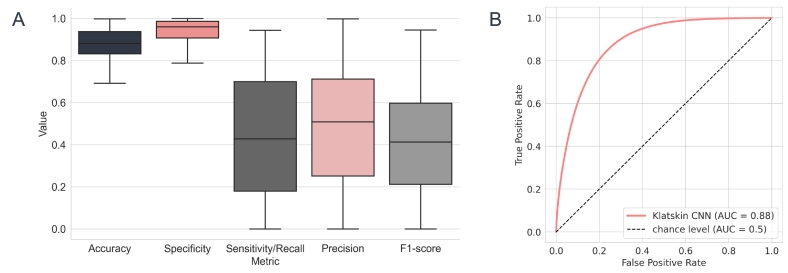
Distribution of accuracy, specificity, sensitivity/recall, precision, and F1-score for each slide image and ROC-AUC curve for all predictions together.

**Fig. 5:**
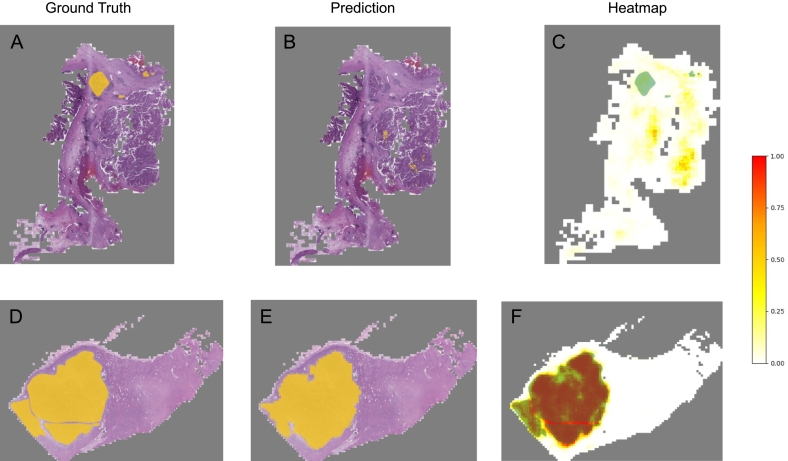
Example of heatmaps. In A–C, a whole-slide image (WSI) with the worst F1-score of 0.0 and in D–F, a WSI with the best F1-score of 0.94. A and D are the ground-truth WSI as annotated by the pathologist, in B and E, the prediction of the model where the probabilities are greater than or equal to 0.5, and in C and F, the heatmap in a white (low) to red (high) scale, with the ground-truth superimposed in green.

On the test set of 142 whole-slide images, the CNN network achieved a specificity of 0.921 (95% CI 0.921, 0.922), sensitivity/recall of 0.534 (95% CI 0.533, 0.534), precision of 0.547 (95% CI 0.547, 0.548), and accuracy of 0.863 (95% CI 0.863, 0.863). In Fig. 4, the distribution of these metrics according to the WSIs is presented. Due to the imbalance in the data with a majority of negative examples, the accuracy and the specificity metrics may not accurately reflect the performance of the model. In spite of these clearly limited metrics, heatmaps were generated from the probability output of the CNN network with the general intention to support the pathologist in the diagnosis of this challenging diagnosis. In Fig. 5, an example of the best- and worst-performing WSI is shown. The obvious limitations of these heatmaps challenge clinical application. The current model would need intense review by an experienced pathologist and could only serve as a subtle guide during diagnosis. However, to improve the utility of the heatmaps, a new training in a larger dataset with accurate tumor cell labeling seems necessary.

### Predictors of postoperative survival

Predictive factors of postoperative survival were studied by uni- and multivariable cox regression analysis. We identified the transfusion requirement during surgery, as well as T- and G grading as independent predictors of survival after surgical resection (Table 4, Table 5).

**Table 4:** Results of univariable cox regression analysis for patient survival (without IHM).

Clinical parameter	*P*-value	Risk ratio	Lower 95% CI	Upper 95% CI
Duration from diagnosis to surgery	.08	1.01	0.99	1.01
Age	.38	0.99	0.97	1.01
Sex	.45	1.21	0.74	1.98
Weight	.11	0.99	0.97	1.01
Height	.57	0.99	0.96	1.02
BMI	.12	0.96	0.91	1.01
ASA Classification	.39	1.28	0.73	2.23
Preoperative Stenting	.74	1.11	0.61	1.99
ASS medication	.12	1.48	0.91	2.39
CA 19-9 preoperatively	.72	1	1	1
Bilirubin preoperatively	.29	1.04	0.97	1.10
Creatinine preoperatively	.99	0.99	0.52	1.89
INR preoperatively	.61	1.62	0.39	6.73
Platelets preoperatively	.47	0.99	0.99	1.001
gGT preoperatively	.09	1	0.99	1
MELD score preoperatively	.18	1.05	0.98	1.12
Duration of surgery	.57	1.001	0.99	1.003
RBC Transfusion	.01	2.76	1.25	6.09
Portal reconstruction	.26	1.62	0.69	3.76
T	.03	1.5	1.03	2.18
G	.001	2.3	1.45	3.66
Lymph node status positive	.065	1.1	0.43	2.41
Lymph node count	.49	1.02	0.97	1.07
L	.88	1.05	0.59	1.85
V	.35	1.42	0.68	2.97
Pn	.02	2.38	1.15	4.89
R	.58	1.11	0.77	1.61
R0 (negative resection margin)	.35	1.25	0.79	1.98
R0 & negative lymph node status	.11	1.48	0.92	2.39
Liver failure (ISGLS)	.32	1.26	0.8	1.98
Re-laparotomy for complications	.58	1.18	0.66	2.09
Bile leak	.08	1.78	0.93	3.38
Postoperative fluid collection/abscess	.73	1.14	0.55	2.37
Postoperative bleeding	.98	1.02	0.25	4.16
Postoperative kidney failure	.76	1.2	0.38	3.81

**Table 5:** Results of multivariable cox regression analysis for patient survival (without IHM).

Clinical parameter	*P*-value	Risk ratio	Lower 95% CI	Upper 95% CI
Transfusion	.003	3.85	1.56	9.38
T	.007	1.85	1.19	2.89
G	.001	3.89	2.13	7.1

### Predictors of postoperative survival by clinical and histological (AI based) features

**Fig. 6:**
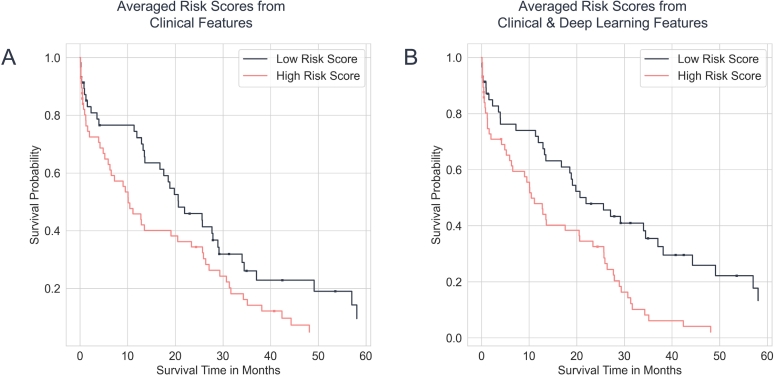
Kaplan–Meier plots for the validation dataset (*n* = 115). The average risk scores produced by the gradient-boosted model are split at the median and divided into a low- and high-risk group. A shows the Kaplan–Meier curves produced with clinical features only, while B shows the curves produced with both deep learning and clinical features.

**Fig. 7:**
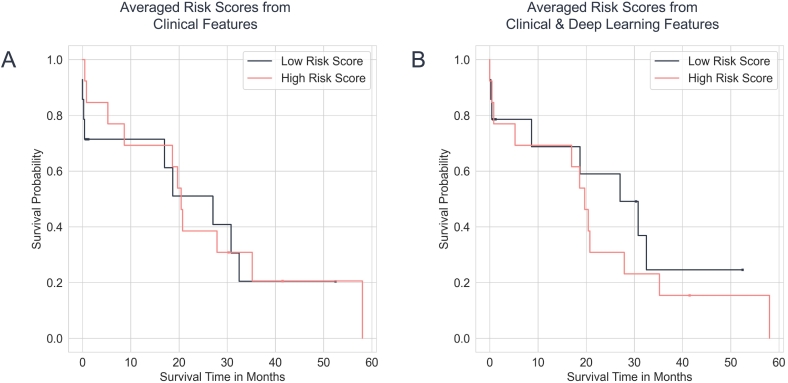
Kaplan–Meier plots for the held-out test dataset (*n* = 27). The average risk scores produced by the gradient-boosted model are split at the median and divided into a low- and high-risk group. A shows the Kaplan–Meier curves produced with clinical features only, while B shows the curves produced with both deep learning and clinical features.

**Fig. 8:**
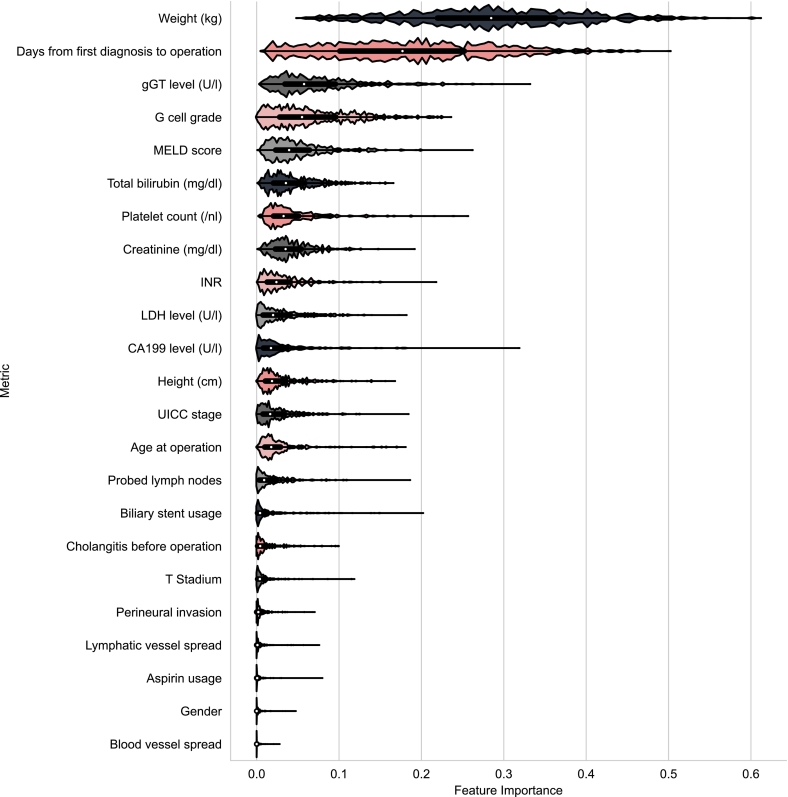
Violin plot of the feature importances of the clinical features. Higher numbers indicate higher importance. The distribution represents the feature importance of the clinical features in the 1000 bootstrapping models. The white dot represents the median.

The risk scores obtained from the machine learning models were used to perform survival analysis on the validation and test sets. For the validation, the clinical features alone reached a rather low concordance index of 0.559 (log-rank *P*-value of .06385). When combined with deep learning features, the concordance index minimally increased to 0.575 (log-rank *P*-value of .00091). On the other hand, for the test set, the clinical features alone gave a concordance index of 0.466 (log-rank *P*-value of .97889). When combined with deep learning features, the concordance index increased to 0.534 (log-rank *P*-value of .38208). The Kaplan–Meier plots are shown in Fig. 6, Fig. 7 for the validation and test set, respectively. Furthermore, the concordance indices of the clinical features computed during the bootstrapping had an average of 0.583 (95% CI: 0.580, 0.587) for the validation set and 0.498 (95% CI: 0.495, 0.500) for the test set. The average concordance index for the clinical features combined with deep learning features model was 0.580 (95% CI: 0.576, 0.583) for the validation set and 0.537 (95% CI: 0.534, 0.541) for the test set. In addition, we also computed the feature importance of the clinical features for the machine learning models. The top 3 most important clinical features for predicting survival were weight, days from diagnosis to operation, and gGT level, which can be reviewed in Fig. 8. This stands in contrast to the cox regression analysis, where these parameters are not significantly associated with the patient survival. Due to the “black-box” nature of CNNs, such difference is not easily explained and resembles different methodological approaches.

## Discussion

We aimed to identify predictive clinical factors in patients with perihilar cholangiocarcinoma and combine these with AI-based histopathological slide analysis for an improved prediction of prognosis.

The present analysis demonstrated as independent predictive factors for the patient survival the T type, grading of the tumor as well as requirements for transfusion during surgery. Both clinical factors have been described before by several studies.^32^, ^33^, ^34^ It is not surprising that tumor biology as represented by the grading of the tumor is a dominant prognosis defining factor. The transfusion requirement, which must be seen in the context of a low transfusion rate of only 10% in the cohort under investigation, also appears to impact outcomes. Usually, intraoperative transfusions may be surrogates for more complex surgery and therefore advanced disease. Interestingly, other studies suggested transfusion-related immunomodulation (TRIM) as a factor compromising immune surveillance, resulting in the elusion of micrometastases.^35^^,^^36^

Unfortunately, the results of this clinical part of our study are representative for other studies^37^, ^38^, ^39^ in that they have a limited ability to predict the individual prognosis.

As such, we aimed to utilize the latest developments in artificial intelligence and digital pathology for a better prediction of prognosis. Digital pathology, fueled by advances in artificial intelligence and computer vision technologies, has emerged in recent years and has already demonstrated the capability of superb prognosis prediction in other tumor entities.^12^, ^13^, ^14^ In the present study, a CNN was trained for whole-slide analysis after marking tumor nests by an experienced hepatobiliary pathologist. Features extracted by the CNN were used for survival analysis after a feature reduction step to ensure the removal of redundant features. This approach was chosen to allow a comparative analysis between the use of solely clinical features and a hybrid of both feature sets. This methodological choice allows for a meaningful comparison between the different feature sources. The comparison of survival prediction by clinical features or combination of both demonstrated that the combination of clinical and histopathological features does not improve survival prediction by the present model (Fig. 6). Unfortunately, the change of the concordance index was small without clinical relevance, as the concordance index of 0.559 (log-rank *P*-value of .064) changed to 0.575 (log-rank *P*-value of .001). From a clinical point of view, the current presented model fails to improve the prediction of the prognosis on an individual basis by digital pathology in perihilar cholangiocarcinoma patients. On the other hand, the presented data demonstrate a first proof of concept that the combination of clinical factors with histopathological features is possible. The very small numerical rise in prognosis prediction might be due to several factors: First, perihilar cholangiocarcinoma present a challenging and variable histopathological morphology, which makes a feature extraction by a CNN challenging, especially with a limited number of slides as in the present study. Second, presumably most important, the current model utilized a marking of tumor areas and tumor nests and not precise marking of tumor cells for the foundation of the CNN-based digital histopathological analysis. As such, feature extraction might have included parts of the slides not containing tumor glands but peritumoral tissue. Furthermore, it is important to acknowledge that the used CNN models inherently lack interpretability,^40^ making it challenging to pinpoint the exact causes of the observed outcomes. To successfully rebuild a model for histopathological-based prognosis prediction in perihilar cholangiocarcinoma, we thrive for a multicenter approach with a precise marking of tumor cells for the foundation of the CNN-based digital histopathologic analysis.

Interestingly, the feature importance of the clinical factors included in the machine learning models disagrees with the classical survival analysis by uni-/multivariable cox proportional hazards. Due to the nature of these models (e.g., black box nature and layer dependency), it remains challenging to explain such differences.

The trained CNN was utilized to generate heatmaps to identify areas of interest in histopathological examinations and hint the pathologists with areas of possible carcinoma cells. In Fig. 5 an example of the best- and worst performing heatmap WSI is shown, demonstrating the applicability and limitations of the model. In the optimal setting all tumor nests are identified and marked up. On the other hand, in some cases, tumor nests are not marked as suspicious, resembling false-negative results, by the CNN, requiring an experienced and attentive pathologist to make the correct diagnosis. Indeed, this is the dominant and clinically relevant limitation: In such cases, a pathologist might be misled and guided to a false feeling of confidence, that nothing is present on the slide, with potentially huge clinical relevance. Accuracy, specificity, recall, precision, and F1 scores, as appropriate metrics to describe the capability of this model, demonstrate that the generated heatmaps are up to now at the most a subtle guiding instrument during the visual exploration of the slides. Beginners in hepatobiliary pathology may benefit from a visual heatmap. Again, we demonstrate a proof of concept, that visual heatmaps are possible for perihilar cholangiocarcinoma, but a clinical application cannot be recommended in the present form. A future redo in a larger dataset with accurately labeled tumor cells hopefully demonstrates better metrics of the models and therefore heatmaps, which a clinical pathologist can rely on.

Of note, this model is not suitable as a stand-alone fully automatic tool for the histopathological diagnosis of cholangiocarcinoma and does not replace the expertise of an experienced hepatobiliary pathologist.

Limitations of the present study include the limited number of patients and the monocentric design. Moreover, the chosen methodological approach to mark all tumor nests, but not single tumor cells seems to prevent a more precise feature extraction for prediction purposes and even more precise heatmap generation.

To overcome these limitations, future investigations should explore alternative methodological approaches for both the annotation process and model development. Potential model improvements include incorporating more sophisticated data augmentation^41^^,^^42^ and normalization^43^^,^^44^ techniques, as well as the pretraining of the CNN on a wider range of histology images^45^ to enhance its ability to capture general structures. Furthermore, due to the low incidence of the disease, multicentric studies are essential for more robust results and generalizability. The knowledge and experience acquired in the course of this study can serve not only as a foundation for our future research but also as a valuable resource for the broader research community engaged in studying this condition.

## Conclusion

In conclusion, our study demonstrates the proof of concept to utilize AI-based digital pathology for prognosis prediction in perihilar cholangiocarcinoma, but so far without clinical relevance. Based on the present methodology, AI-based slide analysis depicts another blind end in the prognosis prediction, hence, alternative methodology should be utilized in future works. Moreover, the presented model might serve as a subtle heatmap builder for a beginner hepatobiliary pathologist, so far without recommendation for regular clinical application.

## Declaration of Competing Interest

The authors declare that they have no known competing financial interests or personal relationships that could have appeared to influence the work reported in this paper.

## Appendix A Supplementary data

Table S1 Count and percentage of missing data for clinical parameters with at least one missing value. Table S1
