# Supplementary material for: AI-based digital histopathology for perihilar cholangiocarcinoma: A step, not a jump
Source: J Pathol Inform. 2023 Nov 5;15:100345. doi: 10.1016/j.jpi.2023.100345 (PMC10698537; doi:10.1016/j.jpi.2023.100345)
Supplement: Table S1 — Count and percentage of missing data for clinical parameters with at least one missing value. [file mmc1.docx]

**Table S1:** Count and percentage of missing data for clinical parameters with at least one missing value.

| **Clinical Parameter** | **Number of Missing Data Points** | **Percentage of Missing Data Points (%)** |
| --- | --- | --- |
| **CA199 level (U/l)** | 79 | 55.6 |
| **LDH level (U/l)** | 75 | 52.8 |
| **Perineural invasion** | 41 | 28.9 |
| **UICC stage** | 30 | 21.1 |
| **Biliary stent usage** | 19 | 13.4 |
| **Weight (kg)** | 14 | 9.9 |
| **Height (cm)** | 14 | 9.9 |
| **Days from first diagnosis to operation** | 11 | 7.7 |
| **Blood vessel spread** | 10 | 7.0 |
| **Lymphatic vessel spread** | 9 | 6.3 |
| **G cell grade** | 5 | 3.5 |
| **T Stadium** | 3 | 2.1 |
